# Challenges experienced in the dental care of persons with special needs: a qualitative study among health professionals and caregivers

**DOI:** 10.1186/s12903-022-02153-x

**Published:** 2022-04-09

**Authors:** Ramaa Balkaran, Talia Esnard, Maureen Perry, Jorma I. Virtanen

**Affiliations:** 1 The University of the West Indies, St. Augustine, Trinidad and Tobago; 2 Institute of Dentistry, University of Turku, Turku, Finland; 3 Arizona School of Dentistry and Oral Health, A.T. Still University, Mesa Arizona, USA; 4 Faculty of Medicine, University of Bergen, Bergen, Norway

**Keywords:** Qualitative research, Barriers, Special care, Dentistry

## Abstract

**Background:**

This study aimed to explore the challenges experienced in dental health care by professionals and caregivers of persons with special needs in Trinidad and Tobago. This research presented results from the first qualitative study which gained insight into the service component of dental care for people with special needs in this country.

**Methods:**

This qualitative study was conducted between March and June 2021. Recruitment of participants used both purposive and snowball sampling. A semi-structured interview schedule was used in the interviews of dentists, (a dental assistant), physicians and caregivers of people with special needs. Narrative inquiry was used in data analysis. The transcripts were individually coded and a follow-up peer debriefing session to cross reference responses and increase the validity of the analysis was performed.

**Results:**

Barriers related to the provision of dental care included readiness of health care professionals, the level of specialized care and the resources required for patient treatment. Caregivers encountered barriers such as cost and lack of accessible dental care for persons with special needs. Possible solutions were education of key stakeholders, policy intervention, advocacy and prevention strategies.

**Conclusions:**

Caregivers and allied health care professions experience multiple barriers when treating patients with special needs. Study participants indicated a need change in the provision and access of dental services for people with special needs. Education of healthcare professionals, improved social policies and health promotion is warranted.

## Introduction

The World Health Organization defined disability and health with respect to multiple dimensions of impairment, activity limitation and participation restrictions that may affect persons differently, even though they have the same disability [1]. While this biopsychosocial view of disability remains critical to the advancement of health care, research on the care received and the challenges experienced in receiving the necessary care remain limited in persons with disabilities (PWDs).

Research in this field however, is particularly needed, given the reduced capability for persons with intellectual disability to provide self-care and by extension their own oral care [2]. While it is clear that oral diseases experienced by PWDs are the same as those without [3, 4] there is limited research on some of the barriers they encounter when utilizing dental services [5]. Recent research on the barriers that caregivers of people with special needs encounter, include, but are not limited to, inaccessibility to buildings or facilities, discrimination by healthcare staff and lack of information, also strengthen the need for this type of research [6, 7]. Furthermore, access to oral healthcare may be affected by both the service provided and the personnel [8]. This inevitably leads to reduced dental care in PWDs.

This issue of the dental care of people with special needs is of particular concern in Trinidad and Tobago, a twin island in the Caribbean, in which approximately 4% (52,000) of the total population of 1.4 million people, have a disability [9]. Lack of accessibility to special needs dental care in the country has led to a greater demand for dental care in this population. Research regarding the challenges of key stakeholders, including health care professionals (HCP) and primary caregivers, in the process of providing dental care is scarce. To the best of the authors’ knowledge there have been no studies conducted locally on this topic. Qualitative research is useful here as it provides a deeper understanding of a social phenomenon based upon in-depth research of a particular experience. A comprehensive study providing an opportunity to better understand these barriers to dental care by people with special needs has been launched in Trinidad and Tobago. The objective of this study was to explore the challenges experienced in dental health care by professionals and caregivers of PWDs in Trinidad and Tobago.

## Materials and methods

### Research design

Narrative inquiry was used to capture the stories of caregivers and HCPs of people with special needs. This was used to denote the accounts of participants with an understanding of the ability of people to tell the stories in ways that make sense to them [10]. Narrative inquiry was used to examine specific instances/critical instances where doctor-patient or other interactions affected the perceptions and experiences of PWDs or for their caregivers. This narrative study also draws on the behavioural model of health services use to give voice to some of the relational and social considerations to be included within the delivery of dental care. While initial applications have used quantitative analysis to assess the relevance of socio-demographic and enabling factors (in the form of income and health density), the relational and social aspect of this behavioural consideration is substantively absent. The unique contribution of this study therefore is in the qualitative exploration of these relational and structural dynamics on the perceptions and experiences of healthcare providers and caregivers. Additionally, it served to contextualize the perceptions and experiences related to these and to depict the key aspects of this process.

### Methods

The principal investigator (PI) solely collected data related to the objectives of the study via semi-structured interviews. Questions were focused on persons with special needs, how they treated within the dental care system, the adequacy and appropriateness of this care, the readiness and knowledgeability of caregivers and medical professions to provide care, the frequency and experiences related to these visits, as well as the challenges that they experience within this process. These questions however were slightly differentiated for caregivers vis-à-vis that of healthcare professionals. The questions were then subjected to the process of ensuring face validity whereby another coauthor, TE, further reviewed the questions for appropriateness and cultural suitability. There was consistency of questions within and across each group. The two groups comprised caregivers and (HCPs) (physicians and dental health care providers (DHCPs)). These interviews lasted approximately one hour, were conducted virtually using the Zoom platform and recorded with the written permission of participants between March to June 2021. Participants were informed of the voluntary nature of the interviews before giving consent. All identifiers were removed from the interviews upon transcription.

### Participants

There were sixteen participants for this study. The use of semi-structured interviewed with these 16 participants allowed for rich and in-depth data, around the perceptions and lived experiences of participants. This number is consistent with the number of participants within in-depth qualitative research (usually 3–12) and was sufficient to achieve the broader aims of this qualitative research. These participants fell into three key groups, namely DHCPs, caregivers, and physicians. In each case, the interviewers collected data for the three categories. Recruitment involved both purposive and snowball sampling. The purposive sampling identified physicians and DHCPs who had experience with patients with special needs as well as caregivers. Snowballing sampling was then used for referrals from both potential colleagues and caregivers who could contribute to the study. Once consented, a day and time for the interview as established. There were no refusals to participate. Table 1 captures the distribution of participants based on demographics. Ethical approval for this research was granted by the local ethics Committee (CREC-SA.0820/03/2021).

**Table 1:** Participants’ demographics

Participants ID	Transcript #	Sex	Ethnicity	Age	Children and ages with/without disability
D1 (dental therapist/assistant)	3	F	M	39	N/A
D2	7	M	IC	53	N/A
D3	9	F	AC	31	N/A
D4	11	F	AC	32	N/A
D5	12	F	AC	48	N/A
CG1 (self-advocate)	2	M	IC	52	None
CG2	1	F	M		2- 1 is 23 with autism, other is 21 without disability
CG3	4	M	AC/M	58	1- 22 with DS
CG4 (2 caregivers)	6	M	1-M	1–65	2–1 is with 27 CP other is 37 without disability
		M	2-M	2–42	2–1 is 8 with CP other is 17 without disability
CG5 (self-advocate with interpreter)	10	Both F	AC	49	None
P1	5	F	IC	35	N/A
P2	8	M	IC	44	N/A
P3	13	F	AC	54	N/A
P4	14	F	O	46	N/A
P5	15	F	IC	70	N/A

Groups of participants: D—Dental professional, CG—Caregiver, P—Physician

Ethnicity of participants: M—Mixed, IC—Indo-Caribbean, AC—Afro-Caribbean, O—Other

### Data analysis

To address the issue of validity, researchers adapted key strategies for securing the trustworthiness; including those of credibility, transferability and confirmability of the data [11]. After transcription via software program (Otter.ai version 2.0), the PI reviewed and edited all transcriptions for accuracy of the recording. Researchers then individually coded the transcripts, cross-referenced responses through a follow up peer debriefing session, to check for the dependability of interpretations. Peer debriefing served as a way to speak to the differing views among three co-authors, where they existed, and to decide on the most appropriate and justifiable interpretation of the data. Researchers also ensured the manual identification of narrative codes to draw on the relational aspects of the experiences related to dental care and then had a discussion of comparative coding with interpretation of possible themes. This also allowed for strengthening of the data interpretation and relatedly the validity of the findings.

Researchers also used this session to draw themes from the findings and to identify negative cases. The negative cases were used to capture diverging views of participants and to ensure that there is analytical rigor. To ensure the transferability of the data, the researchers applied thick description of the data and detailed accounts of verbatim responses to specific issues or themes as they unfolded. These measures, where applied, were seen as critical to the process of aligning the problem being examined, the methods and the analysis of the said data [12].

## Results

The challenges related to the provision of dental care was a central issue within the results. This unfolded as a complex issue with the readiness of HCPs or their ability to provide treatment for patients with special needs. These apprehensions however extended into the inability to provide specified care for these patients and the lack of resources to do so. The following sub-sections expand on these two issues.

### Specificity and inadequacy of care

The data also captured the nuances of providing holistic treatment. At a general level, D1 for instance spoke about the need to consider the spectrum of special needs and the gendered relations between health care providers and patient. D1 noted therefore that “sometimes, …. patients would prefer having females only; some of them would respond to males only. But that would be based on the history of whatever they've been through … or on the spectrum of special needs for the patient…” For CG1 these concerns for the spectrum of disability and the gendered sensitivities were particularly important given that many HCPs still “…operate [as if they are still working from] a textbook [without an understanding that] each patient is different” and with different experiences or circumstances, which collectively may affect how they see and receive the treatment for health care providers.

CG4 reiterated therefore that “[we need] a special area for people with disabilities” that takes into consideration these social, medical and relational issues. CG4 noted therefore that…, “when they come in…[they] meet everybody…in the same situation so [that they] could relate to each other…[to] have a conversation; it makes one another comfortable ….” P4 also concurred:

“I think there should be specialized dental clinics for children with special needs…where they're coming in with children who look like them… [with adequate] time…so that the dentist themselves can feel quietly confident and take their time and do what they need to do with the thoroughness that is required.”

Against the consideration for specialized and holistic care, P1 called for a collaboration to “…include dental and physiotherapy and occupational therapy, speech therapy, medical teams, social workers, psychiatrists, [and] psychologists”. For D5 this level of holistic care was particularly needed to address the overall quality of life for PWDs. D5 reiterated therefore that “…. if you take out …. 10 to 15 teeth, we may get them out of pain, but you don't add to …. their quality of life, in terms of nutrition and wellbeing”. Given these considerations for an extended or specialized service, P5 also suggested that there is a need for much better collaboration and information flow between primary and secondary (tertiary) care needed.

Given the above, P2 suggested that there was a general lack of accommodation for the wide spectrum of children with special needs. D5 highlighted the lack of resources for “everything related to delivering examination and diagnostics, and actually treating patients…. I think that's basically the three avenues… not being able to assess patients properly, and then not being able to deliver the care because of limited things like sedation and general anesthesia.” P3 extended the discussion on the limited treatment options available for PWDs and the concerns for the appropriate of the medical treatment that they receive. In providing some detail, P3 shared this experience:

“.. I had to have an adult with a mental age of about five remove wisdom teeth because they were infected and impacted. The dentist sent me a letter asking me if I felt that this could be done under local anesthetic. And could I prescribe something that could sedate the patient? And clearly the dentist is dangerous. So, I told him that I immediately gave a letter to xx hospital got an appointment…[but] you can't leave that child in pain…. could I give Valium to the child to knock the child out? I was like, but you're going to need to sedate the child properly. And you need to do all four one time.”

In some cases, the interviewees suggested that the use of technology was an option in the possibility of prevention of oral diseases. Yet, there were also concern as to the suitability and efficiency of this option. P3 stated “I don't know how much of an oral examination is possible by telemedicine, but there could be some attempt at doing that from a preventive health, preventive oral health point of view… little dental care packages made available for persons who may be known to the clinic, but unable to come.”

### Challenges for caregivers

Caregivers also presented unique perspectives on some of the drawbacks for accessing quality dental care. This included the cost of the preparation and attention required to receive dental care and lack of social support in the process of accessing dental care for people with special needs. These are discussed in the following subsections.

### Cost requirement and responses

Many of the caregivers spoke to the socio-economic realities that they faced and the related difficulties in securing dental care for their children and relatives. At a central level, this issue of the finances remained a core challenge for caregivers, with related concerns, not just for the coverage of dental related fees, but also for that of transportation and other services needed to support the visit. D5 therefore stated that:

“Finances is a big, big issue, [that is] having enough money to attend clinic, hiring a vehicle if they're wheelchair bound. [it is also about access]. It may sound silly but things that you take for granted [also have financial implications including] being able to afford toothpaste, and toothbrush and mouth rinses and all the fancy things [that are] shown to you [as a] suggest[ion of how] you [can attend to dental needs by using things like] special care brushes and whatever else. Unless [the patient has it], [it is important to] give it to them they’re not going to have access to these things.

Similarly, P3 who viewed transportation as a barrier to accessing dental care.

“Sometimes the lack of access of care within the public health sector, for children healthcare is free. But some person they may be living in such levels of integer that they still can't afford, especially if they are positioned in remote areas where they may have to hire some form of transport exclusively to come to wherever the point of service is so that's sometimes a barrier.”

The issues therefore of access and affordability are related to their socio-economic status and inadvertently, inability to address the dental needs of their children or relatives. P4 reiterated that, “my clientele is really low socio economic; they have a hard-enough time meeting special needs nutrition, rent is a struggle, much less for special nutrition, nutritional needs, much less a visit to the dentist.”

Despite the challenges that they face, participants also stressed on the lack of responsiveness to these. Six interviewees spoke specifically to the lack of accommodation or responsiveness to infrastructural, institutional, and social support needs when responding to PWDs. For most patients, these infrastructural and institutional challenges that extended into long wait time, a lack of accommodation, with heightened periods of frustration. Thus, while dental services remained accessible to persons through the clinic, these caregivers called for greater attention to the type of accommodation and level of institutional support that were provided to support persons with disabilities. For example, CG2 shared:

“A lot of times, people with special needs don't access services, like the typical person. And they may not have a tolerance to wait in clinics, free clinics for a long period of time. And so, I was really happy when you decided to do this because … we have been dealing with clients who trying to access basic services, in the hospital, in the clinics, and so on. A lot of families do not take the children to the clinics, because of the long waiting time. And when they do, they just see the doctor like for 5-10 minutes, and then they probably prescribed or they're not sure what's happening.”

For other participants, this non-response or inadequate response/provisions for PWDs also extended to situations that they face at the level of the society. CG4 therefore stated “there isn't much consideration when it comes to people with disability. In terms of the things like government offices, transport, supermarkets, things like that, there isn't much consideration, it's always an afterthought.”

### Moving forward: call to action

In addressing these issues, participants called for policy interventions with specific attention to the education of key stakeholders, advocacy, and preventative action, discussed below.

Participants also suggested that a national policy is required that prioritizes dental care for PWDs. P2 spoke about the deficiency in the resources needed to support people with special needs.

…. disability services are the last to get attention in terms of resources.” [It would be good] “to have services for kids zero to 18 years in the public service and a mechanism by which if the general dentist is not able to manage the child, they can refer to a special needs dentist in the public system, which is free of charge.”

P3 also spoke about this strategy: “on a national level we're talking about governmental policy and plans that would allow for greater access of children with special need to have equitable access to health care.” P4 further advocated for a national response given that the perception that their concerns go unaddressed. P3 summed this up with the statement that “the only thing in the public sector that they do is pull teeth. Literally. That's it!”.

To address these concerns, caregivers also called for policy interventions with attention to the requirements for health promotion and outreach campaign. The common risk factor approach was also suggested as a solution to health promotion in this population,.CG1 captured this in the reference to:

“…people[‘s] lifestyle…. [where] we have very high [number of people] with non-communicable [diseases] like high blood pressure, diabetes. [These] were very high because of our sugar content intake, carbohydrate intake. And same thing it impacts on the teeth…. I think we need to shift our policy or strategy for the prevention…reduce high sugar, content, food and so on.”

The call in this case was for government to use their legislative and executive power to redirect funding to strategies that support healthy lifestyles at a national level. For most participants however, addressing the issue of health promotion required some measure of advocacy and outreach. P4 suggested …. “going to do home visits to old age homes or homes where there are persons with special needs…would be invaluable to them…even if it's once a year.” These measures unfolded as complimentary mechanisms to support a more systemic approach to health care agendas in Trinidad and Tobago.

In educating/sensitizing DHCPs for people with special needs, participants also noted that information should be available to caregivers to help them improve the lives of their loved ones. For these participants, this required training and education on the requirements for supporting PWDs. P1 for instance raised the issue of the lack of information given to caregivers:

“… because they, they didn't have any access to get proper advice on dental care from when these children were a lot smaller, because I think a lot of them would not need such massive sedation or not need to have such a massive cleaning because trying to get a cerebral palsy child eight years of age to put a toothbrush in their mouth, it's going to feel really weird and the sensory parts of it and all of that, when it could have been introduced at a much younger age and wouldn't be …. that difficult.”

Preventative healthcare was discussed by most participants. CG1 stated that “prevention is better than a pound of cure”. P2 said “we should be very proactive…. I wish that kids can have routine dental checks and visits with our dentist regularly to maintain dental and oral health. So that we can take up our preventative approach to dental can avoid all the …. complications…” Interviewees also called for the training of HCPs to manage people with special needs. For instance, CG5 desired better communication “I would like all dentists to know about the deaf community, the blind and other differently abled and learn sign language.” P4 sought curriculum changes. “I do think the curriculum needs to reflect that, special needs, is a vulnerable population and should be treated as such, I think if our junior dentists are taught from now, then we stand the chance for the future generation.”

To address these infrastructural limitations, health care providers called for greater use of soft skills to connect patients to their HCPs. In that regard, HCPs underscored the importance of empathy. D2 shared:

“A lot more empathy. Maybe if dental students could have a special session …. because going to the dentist is scary. It really is because of some of the stuff, and then the noise, it scary for …. an adult with awareness, much less for somebody who's half blind and is hearing things coming to them….”

D1 spoke to the importance of building trust.

“..it's a challenge to get them inside the clinic, because they are so afraid, or they are not familiar with you…. or just a touch is too much for certain patients, ... a touch of somebody unfamiliar”

Collectively, the discussions reiterated the need for training and educating on specific competencies, skills sets and knowledge requirements to better support PWDs.

## Discussion

This study presented the first insight into the main challenges impacting dental attendance in PWDs which are, dental care that is both accessible, and affordable. An inclusive approach to the definition of PWDs, allowed for multiple subgroups/spectra of disability to be included in this research, which was similar to other literature that assessed the health services for PWDs [13]. The main strength of this research is that it was the first qualitative study that deals with the service component of dental care for people with special needs in Trinidad and Tobago. The study also presents diverse accounts of the experiences and challenges with the service aspects of this process from HCPs, self-advocates, and caregivers. The international literature also is limited in that regard.

From the caregivers’ perspectives, the study also confirmed the relevance of the social and relational aspects of dental care for PWDs. Key concerns for participants were therefore the lack of social support to address their already disadvantaged social and economic circumstances including the lack of transportation, lack of wheelchair access, or special institutional arrangements to cater to the needs of persons with special needs. The connection of these institutional factors to dental care is also similar to other literature [6, 7, 14]. Caregivers added to this understanding with specific attention to the perceived lack of experience of the dentist and dental staff to treat patients with special needs. While these findings are consistent with existing research that highlights the need to better prepare dental students by the increased time spent with patients with disabilities [15], participants also called for the inclusion of soft skills to enhance the interactions and interrelations between patient-healthcare provider. These soft skills were perceived to be relevant particularly given the lack of respect for persons with disabilities and a related lack of sensitivity to the unique medical and social circumstances that they face. In this way, the caregivers underscored the importance of not just the social and the behavioral aspects of dental care, but also, that of institutional factors (such as infrastructure and capacity), which also impact both the quality/specificity of care received.

From the HCP, the findings also call for greater considerations of the knowledge, training, and capacity to design and delivery specific dental services for PWDs. These calls were based on the stories of dentists who had negative experiences themselves, and who, although they were willing to treat patients with disabilities, generally felt that they either lacked the knowledge or tools to perform the treatment safely in either the public or private setting. This discomfort by dentists in the treatment of patients with special needs was also found in recent literature [5, 7, 16]. Additionally, dentists discussed the lack of cooperation of patients with disabilities and the reduced treatment options which were available. Similarly, other research has highlighted the correlation between tooth loss and lack of ability to cooperate with the dental treatment [17]. Increased tooth loss was also discussed by participants in this study with respect to the resulting reduced quality of life. Furthermore, our findings emphasized the need for dentists to be trained to communicate with PWDs at both the undergraduate and postgraduate levels in a more sensitive manner [7, 18, 19]. Many HCPs were also appreciative of the societal issues that some patients with disabilities have and often stated that they attempted to alleviate pain before they referred them for specialist care. The current COVID-19 pandemic highlighted the need to meet the requirements of patients with special needs. The special needs dental clinic at the school was closed for almost one year and most private dental clinics were mandated by the government of Trinidad and Tobago to only perform emergency dental work at the beginning of the pandemic [20]. This disproportionately affected the dental attendance of people with special needs both at the dental school and in private practice, during the past year.

At a broader level, the inequalities in the delivery of oral healthcare have been shown in the barriers faced when accessing dental care at the primary level which has led to both the prevalence of disease and unmet dental needs of PWDs [5, 21]. These findings provide critical insights into the design and implementation of dental services to address these social, institutional and relational issues affecting the dental treatment of PWDs. Our research also highlighted several barriers at the national level, which require policy changes at the governmental level, such as grants, physical access and social support for this vulnerable group and their caregivers. In the case of Trinidad and Tobago, while there is free dental health; the services are limited and often do not accommodate people with special needs. The pandemic has undoubtedly intensified some of these concerns related to access. Participants signaled therefore the need for a multilayered approach, which is rooted in behavior change strategies, but starts with public policy initiatives. Central to this behavior change approach is that of the training of healthcare providers, caregivers, and the wider public, with the knowledge and competencies needed to better integrate PWDs. One way to improve the level of professionalism is via service-learning with patients with special needs [15, 16, 22]. Our research reaffirmed the need for more outreach projects with dental teams in the community to improve both the perceptions and experiences with the services for people with special needs. This is especially important given that caregivers play an important role in the prevention of dental disease through improved oral hygiene techniques [17]. Our study also supports the need for clinics that treat people with special needs to collaborate to both identify and determine the practical and personal requirements of this population [14, 23]. The physicians who were interviewed expressed the idea that a top-down approach could facilitate increased access to health care by people with special needs and highlighted the need for a multidisciplinary team approach. This suggestion was similar to other research that called for interprofessional collaboration in the healthcare of PWDs [8, 24]. Research has also advocated for improved use of publicly-funded dental services that have been integrated with other services that benefit this population [16, 25]. The findings underscore the critical role of the state in this process.

### Limitations

There were a few limitations to this study related to the use of online interviews and of HCPs, of caregivers located in one public dental care facility within Trinidad and Tobago, and the limited insights from PWDs. In the first instance, these interviews were conducted online instead of the face-to-face method, due to the pandemic, which may have a different outcome, given the online platform. Although face-to-face interaction is commonly preferred, the interviewees may have felt more relaxed in their own environment to discuss the issues. Additionally, this mode of communication was welcomed by most caregivers, given the complexity of their day-to-day activities, which have now been increased as a result of the recent pandemic. The data analysis strategies (related to debriefing between co-authors with independent and collective coding as a way to triangulation interpretations of the data); a strategy which allowed for a cross-examination of researchers’ questioning, interpretation and conclusions drawn from the data [26, 27].

Secondly, while this study presented new insights into dental care for special needs patients in Trinidad and Tobago, there was a lack of diversity with the selection of HCPs to capture differences in training and experiences. As a major public health care facility, which provides free dental services to citizens of Trinidad and Tobago, the findings however provide important baseline data for building an understanding of the present state of dental care within the public health care system and for expanding on these findings to other public and private health care facilities within the country. Future research can therefore consider a more diverse pool of health care workers and caregivers. The data also suggests the need for further exploration of the training, resources, and perceptions of health care providers, both within the delivery of dental care and the type of outreach that are created to address vulnerable groups of persons within the broader society. The latter was similar to another research that underscored the need to both plan and deliver dental services that meet the needs of PWDs [13].

Thirdly, there was limited data/perspectives from participants with special needs, with only two persons who considered themselves advocates for PWDs. With appropriate tools or methods future research can include the voice or experiences of persons with different special needs from those who participated in this research. The findings also point to broader structural and social challenges that impact the quality and specificity of dental services for PWDs. It is important in that regard for future explorations of social inequalities and its impact of these populations and the equitable ways in which they are being or can be addressed.

## Conclusions

The study addressed challenges related to the dental care of PWDs. This qualitative study presented diverse insights from healthcare providers and caregivers with examinations of the social and behavioral aspects of dental care. The findings however suggest that while the behavioral model provides instructive insights into the dynamics of dental care for PWDs, institutional issues related to the specificity of the service, the quality of the care provided, and the treatment of the HCPs all serve as critical aspects of how PWDs receive dental care. Given the need for more inclusive and equitable agendas in services provided for vulnerable persons, such as those with disabilities, the findings strengthen the call for a national or public policy response with attention to public health education, outreach, and training of healthcare providers.

## Data Availability

The datasets used and/or analyzed during the current study are available from the corresponding author on reasonable request.

## Abbreviations

PWDs: People with disabilities
HCP: Health care professionals
PI: Principal investigator
DHCPs: Dental health care providers
D: Dental professional
CG: Caregiver
P: Physician
M: Mixed
IC: Indo-Caribbean
AC: Afro-Caribbean
O: Other
